# The Impact of the COVID-19 Pandemic on Alzheimer's Disease and Other Dementias

**DOI:** 10.3389/fpsyt.2021.703481

**Published:** 2021-07-14

**Authors:** Jinghuan Gan, Shuai Liu, Hao Wu, Zhichao Chen, Min Fei, Junying Xu, Yuchao Dou, Xiaodan Wang, Yong Ji

**Affiliations:** ^1^Department of Neurology, Beijing Tiantan Hospital, Capital Medical University, Beijing, China; ^2^Department of Neurology, Tianjin Huanhu Hospital, Tianjin, China; ^3^Tianjin Key Laboratory of Cerebrovascular and of Neurodegenerative Diseases, Tianjin Dementia Institute, Tianjin, China; ^4^Graduate School of Tianjin Medical University, Tianjin, China; ^5^Department of Neurology, Yuncheng Central Hospital of Shanxi Province, Shanxi, China; ^6^Department of Neurology, Tianjin Baodi People's Hospital, Tianjin, China; ^7^China National Clinical Research Center for Neurological Diseases, Beijing, China

**Keywords:** Alzheimer's disease, COVID-19, cognitive, dementia, neuropsychiatric symptom

## Abstract

**Introduction:** Numerous countries went into lockdown to contain the COVID-19 outbreak, which has impeded follow-up of chronic diseases, such as cognitive impairment (CI). Cognitive and neuropsychiatric changes during the COVID-19 pandemic are neglected in China, which is the world's whistleblower. To investigate the cognitive and neuropsychologic changes in CI, as well as the proportions of rapid cognitive decline (RCD) before and during the COVID-19 pandemic to provide clinical evidence for CI intervention during a public health emergency.

**Methods:** We performed a descriptive and retrospective study based on medical records from the memory clinic of Tianjin Dementia Institute collected through face-to-face evaluations. Information of 205 patients with CI, including patients with mild cognitive impairment and dementia, of whom 131 with Alzheimer's disease (AD) were analyzed and compared to a control group before the COVID-19 pandemic.

**Results:** Among the 205 CI patients, the scores on the Chinese Mini Mental State Examination (C-MMSE), the Montreal Cognitive Assessment (MoCA), activities of daily living (ADLs), and the global Neuropsychiatric Inventory (NPI) were significantly different at the baseline and follow-up evaluations (*p* < 0.05) after 14.07 (±2.87) months. The same findings were recorded among AD patients, and they exhibited more sleep disturbances at the follow-up than at baseline (32.8 vs. 20.6%, *p* = 0.035). When compared to the control group, slightly worse performance of cognitive, −1.00 (−4.00, 1.00) from the C-MMSE, −1.00 (−2.00, 0.00) on the MoCA, 1.00 (0.00, 9.00) on ADLs and neuropsychological 0.00 (−1.00, 3.50) on the global NPI profile, at the follow-up were presented, particularly for delusion, agitation, irritability, and appetite disturbances (*p* < 0.05). Twenty-five (19.1%) AD patients and 48 (36.6%) controls suffered RCD during the COVID-19 pandemic. Moreover, AD patients during the COVID-19 pandemic were 0.408 times (95% confidence interval: 0.232–0.716) less likely to suffer RCD than the control.

**Conclusion:** Confinement might ease the cognitive and neuropsychiatric deterioration of AD patients compared to those not in crisis and help prevent RCD in AD patients.

## Introduction

In December 2019, the coronavirus disease 2019 (COVID-19) outbreak caused by the novel coronavirus SARS-CoV-2, which primarily affects the lower respiratory tract, occurred, and aggressively spread around the world (1). To fight the pandemic and limit the spread, many governments, including the Chinese government were obliged to impose a variety of “lockdown” measures in January 2020. By December 2020, COVID-19 has caused a 1-year pandemic affecting the lives and livelihoods of the entire human population.

According to some studies, COVID-19 is more severe in older adults (2, 3) and most of whom have comorbidities (4–6). Patients with dementia need long-term treatment and specialized care, and their cognitive and neuropsychological status differ from that of the general population. COVID-19 patients with dementia, particularly in the severe stage, suffer higher mortality than those without (62.2 vs. 26.2%) (7). The double hit of dementia and the COVID-19 pandemic has raised great concern for patients living with dementia. The present report on the prevalence of cognitive impairment (CI) in China shows that the overall prevalence of dementia is 6.0% and that of mild cognitive impairment (MCI) is 15.5%. These proportions represent 15 million patients with dementia and nearly 38 million patients with MCI in China (8). CI has emerged as a pandemic in the aging society.

The apolipoprotein E (ApoE) ε4 genotype is associated with dementia, and the ε4ε4 (homozygous) genotype is associated with a 14-fold increase in the risk of Alzheimer's disease (AD) (9) compared to the common ε3ε3 genotype. Abnormal amyloid-β (Aβ) and tau aggregation are the major features of AD pathology. Furthermore, AD patients with ApoE ε4 undergo an accelerated memory decline. A recent study also showed that the ApoE ε4ε4 allele increases the risk of severe COVID-19 infection, independent of pre-existing dementia, cardiovascular disease, and type-2 diabetes by affecting lipoprotein function (and subsequent cardio-metabolic diseases moderating macrophage pro-/anti-inflammatory phenotypes) (10).

Dementia patients primarily live with their spouses or children, or in nursing homes. The lockdown attempted to limit the spread of COVID-19; thus, patients with dementia and their caregivers stayed at home together. Moreover, patients with dementia may not understand changes in their life and it may be difficult for them to adapt to lockdown because of their disturbed routines. The ability to explain COVID-19 and the lockdown to a patient with dementia depends on disease severity and the patient's need to acclimatize themselves to a new routine. These changes may affect the quality of care, as well as the progress of dementia. The follow-up of chronic diseases, including dementia, has been delayed or relegated in many cases due to the pandemic.

We carried out the first longitudinal study on the consequences of the COVID-19 pandemic on dementia. We followed up patients with dementia who visited the memory clinic of Tianjin Dementia Institute from 1 January to 12 December 2019 and evaluated their cognitive and neuropsychological profiles face-to-face during the COVID-19 pandemic to investigate cognitive and neuropsychologic changes, as well as the proportion of rapid cognitive decline (RCD) during the COVID-19 pandemic. These findings provide clinical evidence for CI interventions during a public health emergency.

## Materials and Methods

### Participants

A total of 436 subjects were seen by a CI specialty clinical service at the memory clinic of Tianjin Dementia Institute, Tianjin Huanhu Hospital from 1 January to 12 December 2019. Among them, 332 patients were given a definitive diagnosis. A two-specialist panel was used to confirm the diagnoses. If there was disagreement, the subject was excluded (*n* = 104). The panel was diagnosed based on the corresponding diagnostic criteria; the MCI diagnostic criteria were based on the International Working Group's description (11). Dementia was diagnosed according to the criteria outlined in the Diagnostic and Statistical Manual of Mental Disorders-Fourth Edition (12). AD was based on the National Institute on Aging-Alzheimer's Association criteria by McKhann et al. in 2011 (13). Blood tests, the ApoE genotypes, neuroimaging (including CT scans and MRI), and positron emission computed tomography were performed, if necessary, to make the diagnosis (14). Twelve patients were diagnosed with mixed or secondary dementia, 61 patients were lost to follow-up and 54 patients had stopped antidementia drug therapy for more than 1 week and were excluded. Finally, 205 participants with CI, of whom 131 had AD, 14 had MCI, and 60 had other dementias, including vascular dementia (15), frontotemporal lobe dementia (16), dementia with Lewy bodies (17), and Parkinson's disease with dementia (18), had at least the first follow-up and continuous antidementia drug therapy records between 1 April and 30 November 2020 (during the COVID-19 pandemic) and were enrolled in this study.

To explore the correlation between the COVID-19 pandemic and RCD, we strictly selected 131 age-, gender-, educational-, course-, and severity-matched AD patients as a control group, who visited the same memory clinic from 1 January 2017 to 31 December 2018 as a control group before the COVID-19 pandemic, and experienced an average of 13.63 (SD = 0.81, *p* = 0.178) months of follow-up. All controls were treated with antidementia drugs, and no differences in the drugs were observed among participants during the COVID-19 pandemic. The same information was collected from the controls.

This study was designed and conducted in accordance with the Declaration of Helsinki, and written informed consent was obtained from all participants.

### Assessment

#### Neuropsychological Measurements

We reviewed 205 face-to-face evaluation records during the COVID-19 pandemic, with an average of 14.07 months follow-up. Demography, medical history, and a neuropsychological evaluation that included the Chinese Mini-Mental State Examination (C-MMSE) (19), the Montreal Cognitive Assessment (MoCA) (20), activities of daily living (ADL) (21), the Neuropsychiatric Inventory (NPI) (22), and the etiological data at baseline and follow-up were reviewed. The Clinical Dementia Rating Scale (CDR) (23) was used to assess the severity of CI as 0.5, 1.0 (mild), 2.0 (moderate), or 3.0 (severe). RCD due to AD was defined as a loss of ≥3 C-MMSE points at the 12-month follow-up assessment (24).

#### ApoE Genotyping

Genomic DNA was extracted from whole peripheral blood, and the ApoE gene was amplified by polymerase chain reaction (PCR) (25). The PCR primers were: 5′-TCCAAGGAG-GTGCAGGCGGCGCA-3′ (upstream) and 5′-ACAGAATTCGCCCCGGCCTGGTACACTGCCA-3′ (downstream). Each amplification reaction contained 200 ng of genomic DNA, 25 pmol of the primers, 2.5 μl of 10% dimethyl sulfoxide, and 0.5 units of Taq DNA polymerase in a final volume of 25 μl. The thermal reactor was programmed as follows: initial denaturation at 94°C for 5 min, 40 cycles at 94°C for 1 min, annealing at 65°C for 1 min, extension at 72°C for 1 min, and final extension at 72°C for 10 min. The amplification product (20 μl) was digested with 5 units of Cfo1 for at least 3 h at 37°C. The samples were resolved by 12% native polyacrylamide gel electrophoresis for 2 h at 200 V. The gels for patient genotyping were stained with 0.5 μg/ml ethidium bromide, and DNA sizes were determined by imaging under ultraviolet light. We determined all genotypes without knowledge of the patient/control status.

#### PET Imaging

^11^C-Pittsburgh compound-B (PIB) PET and ^18^F-AV45 PET scans can be used to evaluate Aβ deposition (26). Patients were diagnosed with Aβ deposits (positive) based on both visual interpretations of elevated binding in the neocortex and semi-quantitative PIB-positive assessments (SUVR > 1.40 for; SUVR > 1.11 for AV45-positive).

### Statistical Analysis

Quantitative variables (age, courses, scores on the C-MMSE, MoCA, ADL, global NPI, CDR, and the follow-up and COVID-19 pandemic intervals) are presented as mean ± standard deviation (SD) when the data were normally distributed and the median (Q_25,75_) for non-normally distributed data. Categorical data (education, marriage status, and RCD) are presented as frequency counts and percentages. Student's *t-*tests were used for the normally distributed AD data of the COVID-19 pandemic confinement and control groups, and the Mann-Whitney *U-*test was used for non-normally distributed data.

We compared baseline and follow-up data during the COVID-19 pandemic in all patients. The chi-square test was used to assess differences between the baseline and follow-up on the global NPI. The Wilcoxon signed-rank test was used to compare the scores on the C-MMSE, MoCA, NPI, and ADL at baseline and follow-up. A logistic regression analysis was performed to explore the correlation between the COVID-19 pandemic and RCD.

All data were descriptively analyzed using SPSS version 25.0 software (SPSS 25.0; IBM, Armonk, NY, USA). A *p*-value < 0.05 was considered significant.

## Results

### Changes in Cognitive and Neuropsychological Symptoms

A total of 205 patients (103 females; mean age = 70.62 years) were included in this study, and their baseline characteristics are shown in Table 1. The majority (131, 63.9%) of the participants were diagnosed with AD, with a mean course of 52.67 (SD = 30.87) months and the highest proportion of Aβ deposition (95.1%). The mean duration of confinement of the 131 AD patients was 8.89 months (SD = 1.91). As shown in Table 2, no significant differences in the CDR scores or the proportions of neuropsychiatric symptoms were found between the initial and final evaluations. The scores on the C-MMSE, MoCA, ADLs, and global NPI were significantly different between the baseline and follow-up evaluations (*p* < 0.05) after almost 14 months. The same findings were observed in AD patients, but AD patients had more sleep disturbances at follow-up (*p* = 0.035).

**Table 1:** Baseline characteristics of the 205 patients with CI.

	**Overall (*n* = 205)**	**AD (*n* = 131)**	**ODs (*n* = 60)**	**MCI (*n* = 14)**
**Gender (*****n*****, %)**				
Male	102 (49.8)	61 (46.6)	35 (58.3)	6 (42.9)
Female	103 (50.2)	70 (53.4)	25 (41.7)	8 (57.1)
**Age, mean (SD)**	70.62 ± 7.96	70.81 ± 7.54	71.15 ± 7.75	66.64 ± 11.60
**Years of education (*****n*****, %)**				
0	12 (5.9)	8 (6.1)	4 (6.7)	0 (0.0)
1–6	32 (15.7)	20 (15.4)	11 (18.3)	1 (7.1)
7–9	55 (26.8)	42 (32.3)	12 (20.0)	1 (7.1)
10–12	61 (29.8)	30 (23.1)	24 (40.0)	7 (50.0)
13 +	44 (21.5)	30 (23.1)	9 (15.0)	5 (35.8)
**Marriage (*****n*****, %)**				
Married	162 (79.0)	104 (79.4)	47 (78.3)	11 (78.6)
Single	2 (1.0)	1 (0.8)	1 (1.7)	0 (0.0)
Divorced	4 (2.0)	2 (1.5)	1 (1.7)	1 (7.1)
Widow	37 (18.0)	24 (18.3)	11 (18.3)	2 (14.3)
Remarried	0 (0.0)	0 (0.0)	0 (0.0)	0 (0.0)
**Living states (*****n*****, %)**				
With spouse	156 (76.1)	103 (78.6)	44 (73.3)	9 (64.3)
With children	28 (13.7)	15 (11.5)	11 (18.3)	2 (14.3)
Alone	14 (6.8)	8 (6.1)	4 (6.7)	2 (14.3)
Others^a^	7 (3.4)	5 (3.8)	1 (1.7)	1 (7.1)
**Courses of disease, mean (SD)**	48.61 ± 31.18	52.67 ± 30.87	43.70 ± 31.00	31.71 ± 27.99
**Smoking, yes (*****n*****, %)**	55 (26.8)	34 (26.0)	19 (31.7)	2 (14.3)
**Alcohol consumption, yes (*****n*****, %)**	36 (17.6)	23 (17.6)	11 (18.3)	2 (14.3)
**DM, yes (*****n*****, %)**	31 (15.1)	19 (14.5)	10 (16.7)	2 (14.3)
**Hypertension, yes (*****n*****, %)**	72 (35.1)	46 (35.1)	23 (38.3)	3 (21.4)
**Heart disease, yes (*****n*****, %)**	12 (5.9)	8 (6.1)	2 (3.3)	2 (14.3)
**Stroke, yes (*****n*****, %)**	21 (10.2)	14 (10.7)	6 (10.0)	1 (7.1)
**Aβ** **deposition (*****n*****, %)**				
Positive	43 (78.2)	39 (95.1)	1 (11.1)	3 (60.0)
Negative	12 (21.8)	2 (4.9)	8 (88.9)	2 (40.0)
**ApoE** **ε4 genotypes (*****n*****, %)**				
ApoE ε4 (+)	13 (23.6)	11 (26.8)	1 (11.1)	1 (20.0)
ApoE ε4 (–)	42 (76.4)	30 (73.2)	8 (88.9)	4 (80.0)

a *Others means living with other relatives, friends, or in nursing home. CI, cognitive impairment; AD, Alzheimer's disease; ODs, other dementias; MCI, mild cognitive impairment; SD, standard deviation; DM, diabetes mellitus; Aβ, Amyloid β; ApoE, Apolipoprotein E*.

**Table 2:** Changes in neuropsychiatric performance during COVID-19 pandemic confinement.

	**Overal**			**AD**		
	**Baseline**	**Follow-up**	***p*-value**	**Baseline**	**Follow-up**	***p*-value**
**Follow-up Interval, mean (SD)**	14.07 ± 2.87			13.95 ± 2.61		
**Confinement Interval, mean (SD)**	9.01 ± 1.82			8.89 ± 1.91		
**CDR, (*****n*****, %)**	1.81 ± 0.89	1.83 ± 0.91	0.038	1.92 ± 0.82	1.91 ± 0.83	ns
0.5	20 (9.8)	27 (13.2)	ns	-	7 (5.3)	ns
1	67 (32.7)	55 (26.8)	ns	49 (37.4)	38 (29.0)	ns
2	60 (29.3)	63 (30.7)	ns	43 (32.8)	49 (37.4)	ns
3	58 (28.3)	60 (29.3)	ns	39 (29.8)	37 (28.2)	ns
**C-MMSE, mean (SD)**	16.50 ± 8.16	14.96 ± 9.02	<0.001	15.64 ± 7.32	14.24 ± 8.15	<0.001
**MOCA, mean (SD)**	12.60 ± 7.54	11.47 ± 8.18	<0.001	11.76 ± 6.84	10.65 ± 7.41	<0.001
**ADL, mean (SD)**	30.97 ± 13.60	36.67 ± 18.61	<0.001	31.06 ± 13.31	37.40 ± 18.12	<0.001
**NPI, (*****n*****, %)**	8.15 ± 10.35	10.40 ± 12.70	0.001	7.27 ± 9.61	9.63 ± 12.37	0.028
Delusions	36 (17.6)	34 (16.6)	ns	20 (15.3)	17 (13.0)	ns
Hallucinations	39 (19.0)	47 (22.9)	ns	21 (16.0)	29 (22.1)	ns
Agitation	53 (25.9)	46 (22.4)	ns	30 (22.9)	25 (19.1)	ns
Depression	56 (27.3)	63 (30.7)	ns	37 (28.2)	36 (27.5)	ns
Anxiety	53 (25.9)	53 (25.9)	ns	32 (24.4)	30 (22.9)	ns
Euphoria	6 (2.9)	10 (4.9)	ns	3 (2.3)	7 (5.3)	ns
Apathy	73 (35.6)	72 (35.1)	ns	48 (36.6)	40 (30.5)	ns
Disinhibition	20 (9.8)	23 (11.2)	ns	9 (6.9)	12 (9.2)	ns
Irritability	70 (34.1)	73 (35.6)	ns	38 (29.0)	42 (32.1)	ns
Aberrant motor behavior	41 (20.0)	48 (23.4)	ns	25 (19.1)	33 (25.2)	ns
Sleep disturbances	57 (27.8)	76 (37.1)	ns	27 (20.6)	43 (32.8)	0.035
Appetite disturbances	39 (19.0)	37 (18.0)	ns	28 (21.4)	25 (19.1)	ns

*CI, cognitive impairment; AD, Alzheimer's disease; SD, standard deviation; CDR, Clinical Dementia Rate; C-MMSE, Chinese Mini-Mental State Examination; MoCA, the Montreal Cognitive Assessment; ADL, the activities of daily living; NPI, the Neuropsychiatric Inventory*.

**Figure 1:**
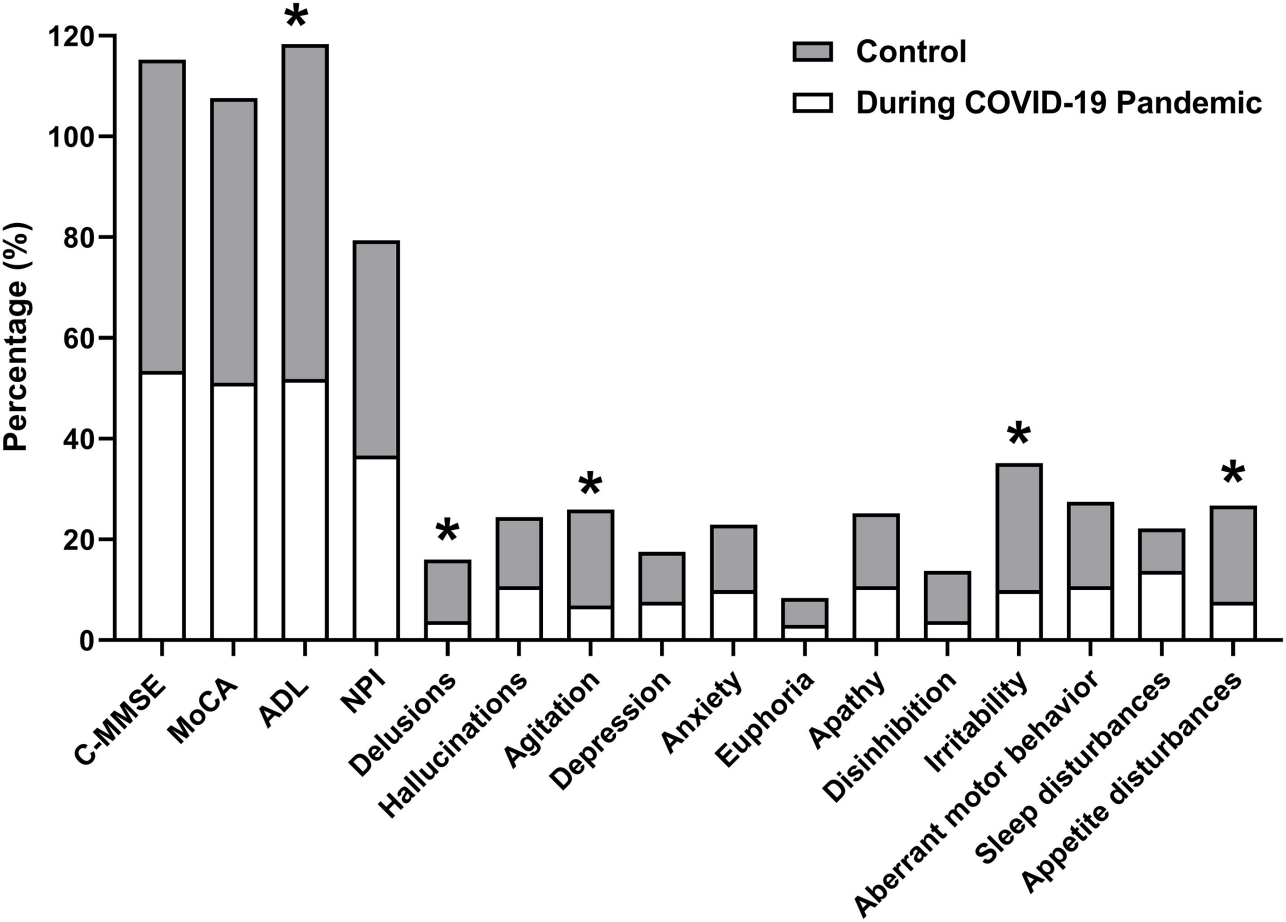
Worsen proportions of cognitive and neuropsychological symptoms during follow-up. The proportions of patients with decreases on the C-MMSE and MoCA scores, and increases on ADL and the NPI (including all items) scores are analyzed and described. C-MMSE, Chinese Mini-Mental State Examination; MoCA, the Montreal Cognitive Assessment; ADL, activities of daily living; NPI, the Neuropsychiatric Inventory. *p < 0.05.

The worsen proportions of cognitive and neuropsychological symptoms during the follow-up are shown in Figure 1. AD patients during the COVID-19 pandemic presented slightly worse cognitive (AD during COVID-19 pandemic vs. control: 53.44 vs. 61.83% in C-MMSE; 51.15 vs. 56.49% in MoCA; 51.59 vs. 66.64%) and neuropsychiatric (36.64% vs. 42.75% in NPI) profiles at the follow-up examination, compared with the control group, but no significant differences were observed except in ADLs (*p* = 0.049). Overall, those who experienced the COVID-19 pandemic had a lower proportion of neuropsychiatric symptoms, particularly delusion, agitation, irritability, and appetite disturbances (*p* < 0.05).

**Figure 2:**
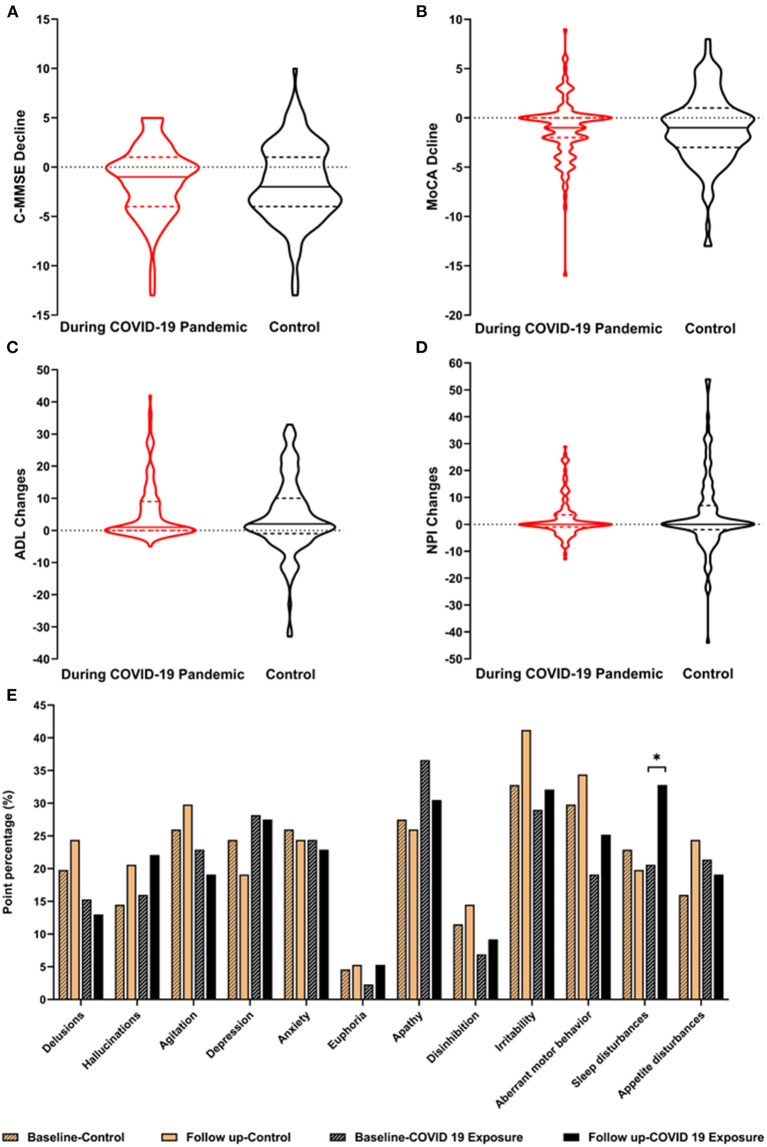
Cognitive and neuropsychiatric changes between the AD groups. The changes in the cognitive and neuropsychiatric scores before and after the COVID-19 pandemic are described in **A–E**. The point percentages of the 12 NPI items are described to show the comparison before (control group) and after COVID-19 (COVID-19 exposure group) at baseline and at the 1-year follow up. AD, Alzheimer's disease; C-MMSE, Chinese Mini-Mental State Examination; MoCA, the Montreal Cognitive Assessment; ADL, activities of daily living; NPI, the Neuropsychiatric Inventory. *p < 0.05.

Figure 2 shows the cognitive and neuropsychiatric changes at baseline and the follow-up between the COVID-19 pandemic and control groups. The median scores on the C-MMSE changed −1.00 (−4.00, 1.00), those on the MoCA changed −1.00 (−2.00, 0.00), ADLs changed 1.00 (0.00, 9.00), and the NPI changed 0.00 (−1.00, 3.50) during the COVID-19 pandemic, but no significant differences in the scores were observed between the two groups. The point proportions of neuropsychiatric symptoms at baseline and follow-up are shown in Figure 2E. The proportions for most symptoms changed similarly in the groups, and hallucinations, euphoria, disinhibition, irritability, and aberrant motor behavior increased from baseline to the final follow-up. Delusions, agitation, sleep disturbances, and appetite disturbances in the patients with AD during the COVID-19 pandemic were opposite in trend compared with the control group. Notably, 20.6% of AD patients during the COVID-19 pandemic developed sleep disturbances at baseline and 32.8% had sleep disturbances at the final examination, which was more frequent than the controls (22.9% at baseline, 19.8% at final examination, *p* < 0.05).

### Association Between RCD and the COVID-19 Pandemic

A total of 73 patients had RCD at the follow-up [25 (19.1%) in the AD group during the COVID-19 pandemic and 48 (36.6%) in the control group]. The proportions of RCD among patients with AD of different severities, ApoE genotypes, and Aβ deposition are presented in Table 3. AD patients in the control group developed RCD more frequently than those during the COVID-19 pandemic, particularly patients with mild (40.4 vs. 18.4%, *p* = 0.017) and severe (35.1 vs. 10.3%, *p* = 0.009) AD. AD patients with the ApoE ε4 allele, and Aβ deposition during the COVID-19 pandemic had a lower proportion of RCD than those in the control group. In this study, AD patients during the COVID-19 pandemic were less likely to have RCD with a risk of 0.408 (95% confidence interval: 0.232–0.716) compared with the control. Mild and severe AD patients were 0.332 and 0.211 times less likely to have RCD, respectively.

**Table 3:** Proportions of RCD in AD patients at the follow-up.

	**With COVID-19 pandemic**		**Control**		**OR, 95%CI**
	**Num**.	**RCD proportion (*n*, %)**	**Num**.	**RCD proportion (*n*, %)**	
**Overall**	131	25 (19.1)	131	48 (36.6)^*^	0.408 (0.232–0.716)^*^
**Initial CDR**					
1	49	9 (18.4)	47	19 (40.4)^*^	0.332 (0.131–0.839)^*^
2	43	12 (27.9)	47	16 (34.0)	ns
3	39	4 (10.3)	37	13 (35.1)^*^	0.211 (0.061–0.726)^*^
**ApoE genotypes**					
ApoE ε4 (+)	11	3 (27.3)	19	8 (42.1)	ns
ApoE ε4 (–)	30	7 (23.3)	33	12 (36.4)	ns
**Aβ** **deposition**					
Positive	39	9 (23.1)	49	20 (40.8)	ns
Negative	2	1 (50.0)	3	0 (0.0)	ns
**ApoE genotypes + Aβ deposition**					
ApoE ε4 (+), Aβ (+)	10	2 (20.0)	19	8 (42.1)	ns
ApoE ε4 (+), Aβ (–)	1	1 (100.0)	0	0 (0.0)	ns
ApoE ε4 (-), Aβ (+)	29	7 (24.1)	30	12 (40.0)	ns
ApoE ε4 (-), Aβ (–)	1	1 (100.0)	3	0 (0.0)	ns

*In this study, only 55 patients, including 41 AD patients, in “during COVID-19 pandemic” group, and 52 patients with AD in control group had ApoE genotyping and Aβ deposition records. RCD, rapid cognitive decline; AD, Alzheimer's disease; Num., number of samples; OR, odds ratio; CI, confidence interval; CDR, Clinical Dementia Rate; ApoE, Apolipoprotein E; Aβ, Amyloid β*.

* *p < 0.05*.

## Discussion

This is the first study on the cognitive and neuropsychiatric symptoms in patients with CI, particularly those with AD, during the COVID-19 pandemic. Among the 205 patients with CI, 131 had AD, and the average scores on the MMSE, MoCA, ADLs, and NPI decreased during the pandemic. More patients with AD during the COVID-19 pandemic had poor ADLs and neuropsychiatric symptoms (delusion, agitation, irritability, and appetite disturbances) compared with the control, but no significant differences were observed in the scores. Also, patients with AD during the COVID-19 pandemic were less likely to have RCD, particularly those with mild and severe AD, compared with the control.

As demonstrated by our study, half of the patients with AD in both groups presented significant cognitive decline over time. Interestingly, COVID-19 confinement resulted in fewer poor cognition cases than controls, even though we did not find differences in the MMSE and MoCA scores between the two groups. Thus, the cognitive differences between the two groups appeared to be intrinsic and not to have been influenced by confinement. Although no significant cognitive changes were observed in Barguilla's study of AD caregivers (27), 60% of CI caregivers perceived worsening cognition during COVID-19 confinement. This may have resulted from more intense observations provided by the caregivers and was affected by caregiver anxiety. The investigation of CI caregiver factors (including cohabitation, care burden, and mental health) has not been completed and more examinations of CI patients are expected. Similarly, although most of China's economic work was carried out by November 2020, COVID-19 must be controlled and prevented.

When comparing the two groups with neuropsychiatric symptoms at follow-up, the point proportion at baseline and the follow-up was not significantly different regardless of the group, except for sleep disturbances in the AD group during the pandemic. This is roughly the same as the follow-up results for 60 CI patients in Spain (27) and suggests that the neuropsychiatric profile worsened globally (*p* < 0.000), as well as appetite (*p* = 0.004). However, not all of the results have been consistent, and some studies that followed up patients with AD during confinement have reported an increase in the occurrence of psychiatric symptoms (28), particularly depression and anxiety (29). In a population-based survey among Chinese workers, epidemic-related factors were significantly associated with 4–5 times higher risk of anxiety and depression symptoms than before the break (30, 31). However, the neuropsychiatric symptoms changed more in the control group. The majority of the controls showed increases in neuropsychiatric symptoms, particularly delusions, insomnia, irritability, and appetite disturbances; thus, suggesting that COVID-19 has slowed down the onset of mental symptoms. These results are encouraging. We suspect it has something to do with the time spent in care during the COVID-19 pandemic or time spent with relatives. Relatives left home less frequently and some children chose to work at home during the pandemic confinement, which increased the time spent caring for AD patients and to some extent alleviated their loneliness and anxiety. A previous study before the COVID-19 pandemic revealed that closer caregiver-care recipient relationships are associated with a 5-point lower NPI score, as well as an increase of 1-point fewer per year (32). Patients with dementia, who are living long-term with a spouse, have significantly lower NPI median total scores than those who live with children or in a nursing home (5.00 with spouse vs. 9.00 with children, 19.50 at nursing home, respectively). These same patients performed well on the MMSE and Alzheimer's Disease Assessment Scale-Cognitive Subscale (33).

However, some neurologists (34–36) have suggested that social distancing measures and diminished physical contact with family and the outside world (e.g., attending neighborhood meetings), as well as social and physical decline, may have increased loneliness and impacted mental health among patients with AD, which is not conducive to improving their cognitive capacity. Therefore, the impact of various lifestyle changes on dementia patients has differed during the COVID-19 pandemic, particularly for patients with different types or severities of dementia. The advantages and disadvantages of lifestyle changes need personal analyses.

Confinement due to the COVID-19 pandemic reduced the risk of RCD in AD patients compared with the control. Previous studies have shown that women (37), lower education (38), psychiatric symptoms (39), the ApoE ε4 allele (40), and positive Aβ deposition (24) are risk factors for RCD. Mild to moderate AD patients, with the ApoE ε4 allele and Aβ deposition are more likely to present with RCD, which was not completely consistent with our results. Previous studies have reported that the prevalence of RCD varies from 9.5 to 54% (24, 41). In our study, patients with mild and severe AD had only about half the risk of RCD during confinement compared with the control (36.6%), with a proportion of 19.1%. We used the definition of 3 points within 12 months between the two groups, and the percentage (36.6%) of RCD in the controls was very similar to that (40.9%; 95% confidence interval, 36.7–45.1) observed in Tchalla's cohort (37), but slightly lower than that of other reports [e.g., 46% in O'Hara et al. (42); 47.9% in Masse et al. (43); and 51.2% in Buccione et al. (44)]. However, the incidence of 19.1% is much lower than that observed in the REAL-FR cohort study (54%) where RCD was defined as loss of 3 points within 6 months (41) and lower than the incidence of RCD in the ELSA cohort (25%), which used loss of 4 points within 6 months. A longitudinal population-based study (CHAP) (45) reported that participants with the APOE ε4 allele are at higher risk of incident AD, and have a greater proportion of RCD than those without the APOE ε4 allele. Aβ deposition has also been demonstrated to be associated with a greater decline in memory in a prospective study (46). The small sample size may have prevented us from detecting the accelerated effect of the ApoE ε4 allele and Aβ deposition on RCD. We suppose that the COVID-19 pandemic played a protective effect on the incidence of RCD. While confinement has dramatically changed most people's daily lives, it may have indirectly changed the RCD risk factors due to AD.

We assume that long-term care during the COVID-19 confinement provided more opportunity for caregivers to detect a change in the condition of the dementia patients and take timely measures to reduce other complications. Secondly, because RCD is associated with psychiatric symptoms (37), cognitive decline and psychiatric symptoms often co-exist in dementia patients, and the overall deterioration of psychiatric symptoms during confinement was lower in AD patients than that in the control group. Therefore, this may be the main reason why RCD was lower in the COVID-19 pandemic group than in the control group. Thus, long-term companionship, as non-pharmaceutical management, played an important role in the treatment of AD during the pandemic. However, individualized coping strategies should be developed for different dementia patients in the future.

The strength of our study includes the assessment of neuropsychological performance using face-to-face interviews of patients with CI, as cognitive and neuropsychiatric changes can be depicted more clearly in this way. Our method was more scientific by setting a strict control group. The long-term neuropsychological impact of the COVID-19 pandemic on dementia patients will provide new evidence for treating AD during future similar crises. The limitations of our study include the relatively small cohort and the lack of relevant reasons for the cognitive changes, such as suffering from other morbidities, exercise, social work, and other circumstances during confinement. The study on caregivers of dementia was not completed, so it could not be used to explain the neuropsychological changes.

## Conclusion

In conclusion, our study has offered helpful insight into the effects of confinement on neuropsychological function in patients with AD during the COVID-19 pandemic. Our study demonstrates that the cognitive and psychiatric symptoms of CI patients, mainly those with AD, tended to deteriorate, and the confinement eased RCD in AD patients, particularly in those with mild and severe AD. This study provides a reference for similar crises and a basis for the formulation of personalized dementia care.

## Data Availability Statement

The raw data supporting the conclusions of this article will be made available by the authors, without undue reservation.

## Ethics Statement

The studies involving human participants were reviewed and approved by The Committee for Medical Research Ethics at Tianjin Huanhu Hospital and the Tianjin Health Bureau. The patients/participants provided their written informed consent to participate in this study. Written informed consent was obtained from the individual(s) for the publication of any potentially identifiable images or data included in this article.

## Author Contributions

YJ designed the study. JG, SL, and HW wrote the report. YD and ZC did the statistical analyses. MF, JX, and XW contributed to the interpretation and discussion of results and reviewed the manuscript. The collaborating authors contributed to the collection of clinical data. All the authors and the collaborating authors contributed to the article and approved the submitted version.

## Conflict of Interest

The authors declare that the research was conducted in the absence of any commercial or financial relationships that could be construed as a potential conflict of interest.

## References

[1] Zhou F Yu T Du R Fan G Liu Y Liu Z. Clinical course and risk factors for mortality of adult inpatients with COVID-19 in Wuhan, China: a retrospective cohort study. Lancet (London, England). (2020) 395:1054–62. 10.1016/S0140-6736(20)30566-3 PMC7270627 32171076

[2] Lloyd-Sherlock P Ebrahim S Geffen L McKee M. Bearing the brunt of covid-19: older people in low and middle income countries. Bmj. (2020) 368:m1052. 10.1136/bmj.m1052 32169830

[3] Mustaffa N Lee SY Mohd Nawi SN Che Rahim MJ Chee YC Muhd Besari A. COVID-19 in the elderly: a Malaysian perspective. J Glob Health. (2020) 10:020370. 10.7189/jogh.10.020370 33214887 PMC7648892

[4] Zhang L Zhu F Xie L Wang C Wang J Chen R. Clinical characteristics of COVID-19-infected cancer patients: a retrospective case study in three hospitals within Wuhan, China. Ann Oncol. (2020) 31:894–901. 10.1016/j.annonc.2020.03.296 32224151 PMC7270947

[5] Fu D Yang B Xu J Mao Z Zhou C Xue C. COVID-19 infection in a patient with end-stage kidney disease. Nephron. (2020) 144:245–7. 10.1159/000507261 32222703 PMC7179522

[6] Wan Y Wu J Ni L Luo Q Yuan C Fan F. Prognosis analysis of patients with mental disorders with COVID-19: a single-center retrospective study. Aging. (2020) 12:11238–44. 10.18632/aging.103371 32561692 PMC7343444

[7] Bianchetti A Rozzini R Guerini F Boffelli S Ranieri P Minelli G. Clinical presentation of COVID19 in dementia patients. J Nutr Health Aging. (2020) 24:560–2. 10.1007/s12603-020-1389-1 32510106 PMC7227170

[8] Jia L Du Y Chu L Zhang Z Li F Lyu D. Prevalence, risk factors, and management of dementia and mild cognitive impairment in adults aged 60 years or older in China: a cross-sectional study. Lancet Public Health. (2020) 5:e661–e71. 10.1016/S2468-2667(20)30185-7 33271079

[9] Farrer LA Cupples LA Haines JL Hyman B Kukull WA Mayeux R. Effects of age, sex, and ethnicity on the association between apolipoprotein E genotype and Alzheimer disease. A meta-analysis. APOE and Alzheimer Disease Meta Analysis Consortium. Jama. (1997) 278:1349–56. 10.1001/jama.278.16.1349 9343467

[10] Kuo CL Pilling LC Atkins JL Masoli JAH Delgado J Kuchel GA. APOE e4 genotype predicts severe COVID-19 in the UK Biobank Community Cohort. J Gerontol A Biol Sci Med Sci. (2020) 75:2231–2. 10.1093/gerona/glaa131 32451547 PMC7314139

[11] Petersen RC Lopez O Armstrong MJ Getchius TSD Ganguli M Gloss D. Practice guideline update summary: mild cognitive impairment: report of the guideline development, dissemination, and implementation Subcommittee of the American Academy of Neurology. Neurology. (2018) 90:126–35. 10.1212/WNL.0000000000004826 29282327 PMC5772157

[12] American Psychiatric Association. Diagnostic and Statistical Manual of Mental Disorders (DSM-IV-TR). (1994).

[13] McKhann GM Knopman DS Chertkow H Hyman BT Jack CR Jr. Kawas CH. The diagnosis of dementia due to Alzheimer's disease: recommendations from the National Institute on Aging-Alzheimer's Association workgroups on diagnostic guidelines for Alzheimer's disease. Alzheimers Dement. (2011) 7:263–9. 10.1016/j.jalz.2011.03.005 21514250 PMC3312024

[14] Liu S Gan J Hu W Wang X-D Zhu H Du X. The clinical characteristics and subtypes of patients with cognitive impairment in memory clinic. J Clin Neurosci. (2020) 82:186–91. 10.1016/j.jocn.2020.10.031 33317730

[15] Román GC Tatemichi TK Erkinjuntti T Cummings JL Masdeu JC Garcia JH. Vascular dementia: diagnostic criteria for research studies. Report of the NINDS-AIREN International Workshop. Neurology. (1993) 43:250–60. 10.1212/WNL.43.2.250 8094895

[16] Hernandez I Fernandez MV Tarraga L Boada M Ruiz A. Frontotemporal Lobar Degeneration (FTLD): review and update for Clinical Neurologists. Curr Alzheimer Res. (2018) 15:511–30. 10.2174/1567205014666170725130819 28745227

[17] McKeith IG Boeve BF Dickson DW Halliday G Taylor JP Weintraub D. Diagnosis and management of dementia with Lewy bodies: Fourth consensus report of the DLB Consortium. Neurology. (2017) 89:88–100. 10.1212/wnl.0000000000004058 28592453 PMC5496518

[18] Emre M Aarsland D Brown R Burn DJ Duyckaerts C Mizuno Y. Clinical diagnostic criteria for dementia associated with Parkinson's disease. Mov Disord. (2010) 22:1689–707. 10.1002/mds.21507 17542011

[19] Folstein MF Folstein SE McHugh PR. “Mini-mental state”: a practical method for grading the cognitive state of patients for the clinician. J Psychiatr Res. (1975) 12:189–98. 10.1016/0022-3956(75)90026-6 1202204

[20] Nasreddine ZS Phillips NA Bédirian V Charbonneau S Chertkow H. The Montreal Cognitive Assessment, MoCA: a brief screening tool for mild cognitive impairment. J Am Geriatr Soc. (2010) 53:695–9. 10.1111/j.1532-5415.2005.53221.x 15817019

[21] Roberts CE Phillips LH Cooper CL Gray S Allan JL. Effect of different types of physical activity on activities of daily living in older adults: systematic review and meta-analysis. J Aging Phys Act. (2017) 25:653–70. 10.1123/japa.2016-0201 28181837

[22] Feghali Y Fares Y Abou Abbas L. Assessment of neuropsychiatric symptoms in dementia: validity and reliability of the Lebanese version of the neuropsychiatric inventory questionnaire. Appl Neuropsychol Adult. (2019). 10.1080/23279095.2019.1670182. [Epub ahead of print]. 31558052

[23] Morris JC. The Clinical Dementia Rating (CDR): current version and scoring rules. Neurology. (1993) 43:2412–4. 10.1212/WNL.43.11.2412-a 8232972

[24] Jia J Gauthier S Pallotta S Ji Y Wei W Xiao S. Consensus-based recommendations for the management of rapid cognitive decline due to Alzheimer's disease. Alzheimer Dement. (2017) 13:592–7. 10.1016/j.jalz.2017.01.007 28238739

[25] Ji Y Liu M Huo YR Liu S Shi Z Liu S. Apolipoprotein? ε4 frequency is increased among Chinese patients with frontotemporal dementia and Alzheimer's disease. Dement Geriatr Cogn Disord. (2013) 36:163–70. 10.1159/000350872 23887281 PMC4068025

[26] Shi Z Fu LP Zhang N Zhao X Liu S Zuo C. Amyloid PET in dementia syndromes: a Chinese multicenter study. J Nucl Med. (2020) 61:1814–9. 10.2967/jnumed.119.240325 32385166

[27] Barguilla A Fernández-Lebrero A Estragués-Gázquez I García-Escobar G Navalpotro-Gómez I Manero RM. Effects of COVID-19 pandemic confinement in patients with cognitive impairment. Front Neurol. (2020) 11:589901. 10.3389/fneur.2020.589901 33329337 PMC7732426

[28] Boutoleau-Bretonnière C Pouclet-Courtemanche H Gillet A Bernard A Deruet AL Gouraud I. The effects of confinement on neuropsychiatric symptoms in Alzheimer's disease during the COVID-19 crisis. J Alzheimer Dis. (2020) 76:41–7. 10.3233/JAD-200604 32568211 PMC9988367

[29] El Haj M Altintas E Chapelet G Kapogiannis D Gallouj K. High depression and anxiety in people with Alzheimer's disease living in retirement homes during the covid-19 crisis. Psychiatry Res. (2020) 291:113294. 10.1016/j.psychres.2020.113294 32763552 PMC7357507

[30] Zhang XR Huang QM Wang XM Cheng X Li ZH Wang ZH. Prevalence of anxiety and depression symptoms, and association with epidemic-related factors during the epidemic period of COVID-19 among 123,768 workers in China: a large cross-sectional study. J Affect Disord. (2020) 277:495–502. 10.1016/j.jad.2020.08.041 32882506 PMC7448744

[31] Huang Y Wang Y Wang H Liu Z Yu X Yan J. Prevalence of mental disorders in China: a cross-sectional epidemiological study. Lancet Psychiatry. (2019) 6:211–24. 10.1016/S2215-0366(18)30511-X 30792114

[32] Vernon EK Cooley B Rozum W Rattinger GB Behrens S Matyi J. Caregiver-care recipient relationship closeness is associated with neuropsychiatric symptoms in dementia. Am J Geriatr Psychiatry. (2019) 27:349–59. 10.1016/j.jagp.2018.11.010 30616905 PMC6812501

[33] Liao ZL Chen Y Tan YF Zhu JP Qiu YJ Lin SS. [Different care models and symptom progression of Alzheimer's disease in China]. Zhonghua yi xue za zhi. (2019) 99:532–6. 10.3760/cma.j.issn.0376-2491.2019.07.011 30786352

[34] El Haj M Jardri R Larøi F Antoine P. Hallucinations, loneliness, and social isolation in Alzheimer's disease. Cogn Neuropsychiatry. (2016) 21:1–13. 10.1080/13546805.2015.1121139 26740416

[35] Simonetti A Pais C Jones M Cipriani MC Janiri D Monti L. Neuropsychiatric symptoms in elderly with dementia during COVID-19 pandemic: definition, treatment, and future directions. Front Psychiatry. (2020) 11:579842. 10.3389/fpsyt.2020.579842 33132939 PMC7550649

[36] Borges-Machado F Barros D Ribeiro Ó Carvalho J. The effects of COVID-19 home confinement in dementia care: physical and cognitive decline, severe neuropsychiatric symptoms and increased caregiving burden. Am J Alzheimers Dis Other Demen. (2020) 35:1533317520976720. 10.1177/1533317520976720 33295781 PMC10623939

[37] Tchalla AE Clément JP Saulnier I Beaumatin B Lachal F Gayot C. Predictors of rapid cognitive decline in patients with mild-to-moderate Alzheimer disease: a prospective cohort study with 12-month follow-up performed in memory clinics. Dement Geriatr Cogn Disord. (2018) 45:56–65. 10.1159/000487938 29684916

[38] Arce Rentería M Vonk JMJ Felix G Avila JF Zahodne LB Dalchand E. Illiteracy, dementia risk, and cognitive trajectories among older adults with low education. Neurology. (2019) 93:e2247–e56. 10.1212/WNL.0000000000008587 31722961 PMC6937498

[39] Sona A Ellis KA Ames D. Rapid cognitive decline in Alzheimer's disease: a literature review. Int Rev Psychiatry (Abingdon, England). (2013) 25:650–8. 10.3109/09540261.2013.859128 24423219

[40] Packard CJ Westendorp RG Stott DJ Caslake MJ Murray HM Shepherd J. Association between apolipoprotein E4 and cognitive decline in elderly adults. J Am Geriatr Soc. (2007) 55:1777–85. 10.1111/j.1532-5415.2007.01415.x 17979899

[41] Soto ME Andrieu S Cantet C Reynish E Ousset PJ Arbus C. Predictive value of rapid decline in mini mental state examination in clinical practice for prognosis in Alzheimer's disease. Dement Geriatr Cogn Disord. (2008) 26:109–16. 10.1159/000144073 18617740

[42] O'Hara R Thompson JM Kraemer HC Fenn C Taylor JL Ross L. Which Alzheimer patients are at risk for rapid cognitive decline? J Geriatr Psychiatry Neurol. (2002) 15:233–8. 10.1177/089198870201500409 12489920

[43] Masse I Bordet R Deplanque D Al Khedr A Richard F Libersa C. Lipid lowering agents are associated with a slower cognitive decline in Alzheimer's disease. J Neurol Neurosurg Psychiatry. (2005) 76:1624–9. 10.1136/jnnp.2005.063388 16291883 PMC1739466

[44] Buccione I Perri R Carlesimo GA Fadda L Serra L Scalmana S. Cognitive and behavioural predictors of progression rates in Alzheimer's disease. Eur J Neurol. (2007) 14:440–6. 10.1111/j.1468-1331.2007.01693.x 17388995

[45] Rajan KB McAninch EA Wilson RS Weuve J Barnes LL Evans DA. Race, APOEε4, and long-term cognitive trajectories in a biracial population sample. J Alzheimer Dis. (2019) 72:45–53. 10.3233/JAD-190538 PMC6914259 31561363

[46] Lim YY Ellis KA Pietrzak RH Ames D Darby D Harrington K. Stronger effect of amyloid load than APOE genotype on cognitive decline in healthy older adults. Neurology. (2012) 79:1645–52. 10.1212/WNL.0b013e31826e9ae6 23071163

